# Running Parameter Analysis in 400 m Sprint Using Real-Time Kinematic Global Navigation Satellite Systems

**DOI:** 10.3390/s25041073

**Published:** 2025-02-11

**Authors:** Keisuke Onodera, Naoto Miyamoto, Kiyoshi Hirose, Akiko Kondo, Wako Kajiwara, Hiroshi Nakano, Shunya Uda, Masaki Takeda

**Affiliations:** 1 Faculty of Education and Welfare, Biwako-Gakuin University, 29 Fusecho, Higashiomi 527-8533, Japan; k-onodera@biwakogakuin.ac.jp; 2 Graduate School of Health and Sports Science, Doshisha University, 1-3 Tataramiyakodani, Kyotanabe 610-0394, Japan; ctvj0002@mail4.doshisha.ac.jp (W.K.); cyhk0003@mail4.doshisha.ac.jp (H.N.); cyhj0007@mail4.doshisha.ac.jp (S.U.); 3 Research Center for Sports Sensing, Doshisha University, 1-3 Tataramiyakodani, Kyotanabe 610-0394, Japan; namiyamo@mail.doshisha.ac.jp (N.M.); kiyoshi.hirose@komatsu-u.ac.jp (K.H.); kondo@kurume-it.ac.jp (A.K.); 4 Department of Production System Engineering and Sciences, Komatsu University, Nu 1-3 Shicyomachi, Komatsu 923-8511, Japan; 5 Faculty of Engineering, Kurume Institute of Technology, 2228-66 Kamitsumachi, Kurume 830-0052, Japan; 6 Faculty of Health and Sports Science, Doshisha University, 1-3 Tatara Miyakodani, Kyotanabe, Kyoto 610-0394, Japan

**Keywords:** sprint running, GNSS, stride length, stride frequency, velocity

## Abstract

Accurate measurement of running parameters, including the step length (*SL*), step frequency (*SF*), and *velocity*, is essential for optimizing sprint performance. Traditional methods, such as 2D video analysis and inertial measurement units (IMUs), face limitations in precision and practicality. This study introduces and evaluates two methods for estimating running parameters using real-time kinematic global navigation satellite systems (RTK GNSS) with 100 Hz sampling. Method 1 identifies mid-stance phases via vertical position minima, while Method 2 aligns with the initial contact (IC) events through vertical velocity minima. Two collegiate sprinters completed a 400 m sprint under controlled conditions, with RTK GNSS measurements validated against 3D video analysis and IMU data. Both methods estimated the *SF*, *SL*, and *velocity*, but Method 2 demonstrated superior accuracy, achieving a lower RMSE (*SF*: 0.205 Hz versus 0.291 Hz; *SL*: 0.143 m versus 0.190 m) and higher correlation with the reference data. Method 2 also exhibited improved performance in curved sections and detected stride asymmetries with higher consistency than Method 1. These findings highlight RTK GNSS, particularly the velocity minima approach, as a robust, drift-free, single-sensor solution for detailed per-step sprint analysis in outdoor conditions. This approach offers a practical alternative to IMU-based methods and enables training optimization and performance evaluation.

## 1. Introduction

Accurate measurement of running parameters such as the step length (*SL*), step frequency (*SF*), and *running velocity* is crucial for analyzing performance in sprinting events [1,2]. These parameters provide valuable insights for training optimization [3]. Traditionally, they have been obtained using two-dimensional (2D) video analysis [4]. However, 2D analysis typically offers only segment-based averages (e.g., every 10–20 m in a 100 m sprint [5] or every 50 m in a 400 m sprint [6]) and fails to capture detailed, step-by-step variations, limiting assessment of fluctuations in acceleration, peak velocity, and asymmetries. Three-dimensional (3D) motion capture is more precise but expensive, complex, and less practical [7].

Wearable sensors overcome these limitations, enabling detailed per-step analysis [8]. For example, studies utilizing smart socks [9] and insole sensors [10,11] have confirmed their ability to accurately capture foot contact timing. However, while these sensors can determine contact timing and estimate the *SL* based on averaged values, they are not capable of conducting a detailed step-by-step analysis. Furthermore, inertial measurement units (IMUs), widely used as wearable sensors [12], effectively detect initial contact (IC) timings and provide continuous temporal data [13]. However, estimating the *SL* from IMUs is difficult due to cumulative errors and drift [14,15], especially at higher speeds [16], and requires complex calibration and data processing [17]. To our knowledge, no prior research has presented a single-sensor wearable solution which reliably captures step-by-step *SL* data throughout a full 400 m sprint.

Real-time kinematic global navigation satellite systems (RTK GNSSs) overcome IMU-related drift issues by providing centimeter-level positional accuracy [18]. Unlike IMUs, which accumulate drift over time, an RTK GNSS directly measures absolute positions in real time, preventing drift-related errors even during extended runs. Previous studies have shown that low-cost RTK GNSS modules, when mounted on the head and operating at 1–5 Hz, improve distance accuracy over standard GPS watches [19]. However, such low sampling rates limit the ability to capture stride-to-stride variations in sprinting. With recent developments enabling sampling rates up to 100 Hz—well above the conventional 10–15 Hz [14]—RTK GNSSs may now allow precise detection of the *SF* and accurate estimation of the *SL*. As the *SL* and *SF* define the *running velocity* (*velocity* = *SF* × *SL*), all key parameters can be derived directly outdoors, free from the constraints of 3D motion capture systems.

In this study, we propose a novel approach to estimating the running parameters for every step across an entire 400 m sprint using a 100 Hz RTK GNSS receiver. This approach enables the calculation of running parameters on a per-step basis, offering a drift-free, single-sensor solution which eliminates the need for additional devices such as IMUs. We explored two theoretical methods for extracting running parameters from the RTK GNSS data, with both grounded in the spring-mass model of running mechanics [20,21]. The first method (Method 1: vertical position minima) leverages the concept of the spring-mass model [20,21], in which the center of mass (CoM) reaches its lowest point mid-stance. Such a head-mounted GNSS approach has been used in other cyclical sports, such as cross-country skiing, to capture precise kinematic patterns [22,23]. These minima can be used to estimate running parameters such as the *SF* and *SL*. This method relies solely on position data—direct outputs from GNSS—making it computationally straightforward. However, left-right asymmetries in sprint running [24,25] may distort vertical displacement patterns, potentially introducing timing errors. Therefore, we propose the second method (Method 2: vertical velocity minima) as an alternative to the first method. This method also draws on the spring-mass model’s principles but targets the velocity profile. As the CoM descends during the flight phase, it reaches a minimum vertical velocity at IC, where the leg acts as a spring-damper system [21,26]. Similar to IMU-based approaches which identify IC events using vertical acceleration zero-crossings [27,28], we detect IC by finding minima in the vertical velocity derived from GNSS data. Although differentiating positions to obtain the velocity may introduce noise, this method’s alignment with a clear biomechanical event could yield more precise *SF* and *SL* estimates.

Thus, this study aims to compare Method 1 (vertical position minima) and Method 2 (vertical velocity minima) to evaluate their accuracy and reliability in estimating running parameters—*SF*, *SL*, and *running velocity*—during each step of a 400 m sprint. As described in the Materials and Methods section, only two subjects were recruited for this study. Therefore, we regard this study as a pilot study. This initial, small-scale approach follows the precedent of other feasibility studies which used limited samples to develop and refine new measurement methodologies [19,29]. Determining which method provides more accurate and reliable metrics is crucial for optimizing RTK GNSS technology for detailed running performance analyses, especially when testing novel devices in real-world contexts.

## 2. Materials and Methods

### 2.1. Participants

Two collegiate athletes who specialize in the 400 m sprint, one male (Subject A, 22 years old, 181 cm, 72 kg) and one female (Subject B, 19 years old, 161 cm, 46 kg), each with at least three years of competitive experience, were recruited as subjects. Both participants are collegiate-level athletes who compete in regional (district) competitions in the 400 m sprint and are members of their university’s athletic team. Before participation, both subjects were informed of the significance and purpose of this study, and informed consent was obtained in compliance with Doshisha University’s ethics review board guidelines (ethics review approval number: 23034).

### 2.2. Experimental Protocol

To compare and validate the GNSS-based estimates against standard references, we employed a 3D camera system, IMUs, and two 2D cameras. Participants performed a maximal 400 m sprint starting from a crouching position using starting blocks in lane 1. Before the sprint, a standardized warm-up lasting approximately one hour was conducted to replicate the athletes’ typical pre-race routines and ensure they reached their maximum competition speed. The warm-up included mobility drills, sprint technique exercises, progressively intensified running, and short rest periods.

Positional data were recorded using a single RTK GNSS receiver system. By attaching the receiver to the runner’s head, we ensured both unobstructed satellite reception and a stable proxy for the body’s center of mass (CoM) motion [30]. This receiver was mounted onto rugby headgear, with the GNSS unit secured in a waist pack [19].

IMUs were affixed above each ankle to validate the *SF* and detect IC events [13]. Since IC events are best detected near the feet, IMUs were placed at the ankles to capture foot-ground contact accurately. Additionally, an IMU was mounted onto the headgear adjacent to the GNSS antenna solely for synchronization purposes. All IMUs were powered on simultaneously using a dedicated remote controller to ensure their internal clocks started nearly simultaneously. For clarity, we define the coordinate axes of each IMU as follows: the *x* axis is oriented mediolaterally (left-right), the *y* axis is oriented anteroposteriorly (aligned with the running or forward-backward direction), and the *z* axis is oriented vertically (aligned with gravity).

Specifically, we conducted 3D video analysis in two track segments—one curved (286–294 m) and one straight (356–364 m)—to account for biomechanical differences in running on straight versus curved paths [31]. Each segment was recorded by two cameras positioned at approximately 30° and 60° angles relative to the running lane, situated outside the runway behind the runner. These cameras captured an 8-m section spanning the full lane width which was 2 m in height. An 18-point calibration check ensured accurate spatial measurements for the *SL*, *SF*, and *running velocity*. To synchronize the GNSS, IMUs, and video data, the participants briefly stood still (1–2 s) in an upright posture before performing a short vertical jump (15–25 cm) in front of each 3D camera—once for the curved section and once for the straight section—immediately before getting into the starting blocks. This approach of using a short vertical jump for synchronization has been adopted in prior studies [32]. During the brief static posture, we estimated the initial orientation of the head-mounted IMU and removed the gravitational acceleration component from the head IMU signals, referencing previous work [33]. Consequently, the head IMU data were transformed into an absolute coordinate system before the jump. The jump created a distinct waveform in the head-mounted IMU’s vertical axis, the GNSS-derived vertical displacement, and the camera-digitized vertical position of the GNSS antenna. We achieved sub-frame synchronization of the timing data by aligning these characteristic waveforms across all devices during post-processing. A similar event-based synchronization strategy has been reported for multiple wearable systems [34].

Additionally, a 2D camera at the start and finish line recorded the total sprint time, and a bicycle-mounted 2D camera, kept about 10 m behind the runner, verified the total step count throughout the run.

### 2.3. Measurement Device

**Figure 1:**
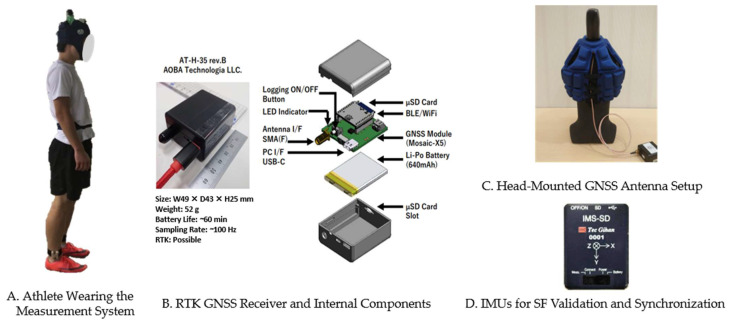
Experimental measurement system. (**A**) Athlete wearing the measurement system, including an RTK GNSS receiver, an antenna, and IMUs. (**B**) RTK GNSS receiver stored in a waist pack. (**C**) Head-mounted GNSS antenna set-up, with a triple-band helical antenna connected via an SMA cable. (**D**) IMUs attached above the ankles for *SF* validation, with an additional unit on the headgear for synchronization.

Figure 1A illustrates an athlete wearing our custom measurement system, comprising an RTK GNSS receiver, a head-mounted GNSS antenna, and multiple IMUs. A GNSS receiver (AT-H-35 rev.B, AOBA Technologia LLC, Sendai, Japan) equipped with a Mosaic-X5 module (Septentrio, Leuven, Belgium) provided RTK positioning at up to 100 Hz (Figure 1B). The Mosaic-X5 supports six satellite constellations (GPS, GLONASS, BeiDou, Galileo, QZSS, and NavIC) and operates on multiple bands (L1C/A, L1C, L2, L5, and L6). The housing of the AT-H-35 rev.B has a volume of 52.7 cm^3^, weighs 52 g, and contains a 640-mAh lithium-ion polymer rechargeable battery, enabling approximately 60 min of continuous operation at 100-Hz sampling. The RTK positioning accuracy is 0.6 cm + 0.5 ppm horizontally and 1 cm + 1 ppm vertically, as specified by the manufacturer [35]. The receiver was carried in a waist pack and connected to a triple-band helical antenna (AS-ANT3B-HEL-L1256-SMA-00, ArduSimple, Lleida, Spain; 40 mm diameter × 82.8 mm length, 35 g) [36] mounted on rugby headgear for satellite signal reception (Figure 1C). The connection was made using an SMA cable, which was secured to the runner’s back with masking tape to prevent interference. All participants wore identical headgear and sensor set-ups for consistency. A stationary reference station near the home straight provided real-time corrections to enhance RTK accuracy [18].

IMUs (IMS-SD, model IMSSD-H-A, Tech Gihan Co., Ltd., Kyoto, Japan; 54 mm × 42 mm × 14 mm) were used for reference *SF* validation and synchronization (Figure 1D). Each unit recorded the tri-axial acceleration (±30 G) and angular velocity (±4000° s^−1^) at 1000 Hz, with data stored internally on microSD cards for post-run analysis. Following the methodology of previous studies [13], each IMU was attached above both ankles, and an additional IMU was mounted near the GNSS antenna on the head to facilitate synchronization.

Four Basler acA1300 cameras (Basler AG, Ahrensburg, Germany), each with an OnSemi PYTHON 1300 sensor (1280 × 1024 pixels), recorded 3D video at 200 fps using StreamPix 8 (NorPix, Montreal, QC, Canada). A GoPro Hero 9 Black (GoPro, Inc., San Mateo, CA, USA) at the start and finish line (120 fps) measured the total sprint time, while another bicycle-mounted GoPro Hero 9 Black followed the runner to verify GNSS-derived step counts.

### 2.4. Data Processing

All data were processed using MATLAB (version 2023a, The MathWorks Inc., Natick, MA, USA). The raw GNSS outputs (vertical position, latitude, and longitude) were sampled at 100 Hz. To remove high-frequency noise while preserving the fundamental signal characteristics related to running kinematics, we applied a zero-phase 4th-order Butterworth low-pass filter with a cutoff frequency of 5 Hz. This cutoff was selected based on a Fourier analysis of the vertical velocity signal, revealing dominant frequency components corresponding to the athletes’ *SF*s. Such a cutoff has been employed in previous studies examining vertical oscillations during running [28]. Filtering was applied identically to the vertical position and horizontal coordinates (latitude and longitude).

**Figure 2:**
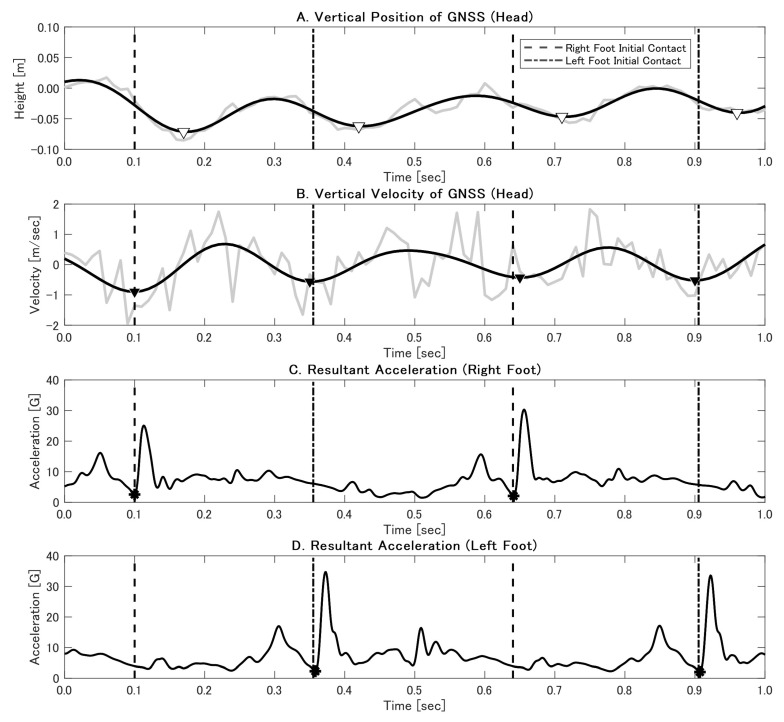
GNSS-based step detection methods with IMU reference. A one-second snapshot of data from Subject A. The dashed vertical lines indicate camera-based IC. (**A**) Method 1 detected minima in vertical GNSS position (▽) near mid-stance. (**B**) Method 2 identified minima in vertical GNSS velocity (▼) aligned with IC. (**C**,**D**) Resultant accelerations from right and left foot IMUs, respectively, with asterisks (*) marking IC events. The grey line shows the raw data before applying the Butterworth filter.

After filtering, we derived the vertical velocity by differentiating the vertical position. Two RTK GNSS–based step detection methods were then implemented. Method 1 (vertical position minima) identifies mid-stance events by finding the minima in the vertical position profile (Figure 2A). The time interval between successive minima defines the step time, from which the *SF* is obtained as *SF* = 1/(*step time*). The horizontal displacement between consecutive minima provides the *SL*, and the *running velocity* is computed as *velocity* = *SL* × *SF*. Method 2 (vertical velocity minima) aligns step detection with an identifiable biomechanical event: IC (Figure 2B). Minima in the vertical velocity trace correspond to IC, allowing direct measurement of step times and subsequent calculation of the *SF*, *SL*, and *velocity*. Both methods rely on continuous GNSS solutions. In this study, the RTK GNSS provided real-time correction data, offering two solution statuses: ‘Fix’ (centimeter-level accuracy) and ‘Float’ (decimeter-level accuracy) [37,38]. Float solutions are primarily caused by incomplete integer ambiguity resolution due to insufficient satellite visibility, signal multipath, or suboptimal environmental conditions [37,38]. Most data were given the ‘Fix’ status, ensuring high positional accuracy. Additionally, segments of ‘Float’ solutions were noted for Subject A.

The IMU data were processed based on methodologies from the previous study [13]. The raw acceleration data from the IMUs were filtered using a zero-phase, 2nd-order Butterworth low-pass filter with a cutoff frequency of 70 Hz. To account for variations in sensor orientation and movement artifacts, we calculated the resultant acceleration by combining the acceleration components from all three axes: $Resultant\;Acceleration=\sqrt{a_{x}^{2}+a_{y}^{2}+a_{z}^{2}}$ , where $a_{x}$ , $a_{y}$ , and $a_{z}$ are the acceleration components along the *x*, *y*, and *z* axes, respectively. Gravity was not explicitly removed from these signals; the resultant acceleration approach inherently accounts for it [13]. IC events were detected using characteristic features in the resultant acceleration signal (Figure 2C,D). The accuracy of the IMU-derived IC timings was confirmed by calculating the mean absolute error (*MAE*) and standard deviation (*SD*) against reference data from the 3D video analysis (200 fps). Consistent with a previous study [13], we excluded the first five steps from further temporal analyses due to the transient mechanics of the initial acceleration phase.

The 3D video analysis provided the primary reference data for the running parameters. The videos were analyzed using WinAnalyze 2.8 software (Mikromak, Berlin, Germany), which is widely used in sports analysis [39,40]. The WinAnalyze system in this experiment demonstrated a median calibration error of 0.017 m with a variance of 0.010 m on the straight path and a median calibration error of 0.015 m with a variance of 0.007 m on the curved path. Initial contact events were identified frame by frame at 200 fps. The *step frequency* was the inverse of the *step time* between successive opposite-foot *ICs*. The *step length* was the horizontal distance between heel positions at these *IC* events, and the *velocity* was *SL* × *SF* [41].

### 2.5. Statistical Analysis

All statistical analyses were performed using MATLAB (version 2023a, The MathWorks Inc., Natick, MA, USA). The significance level was set at *p* < 0.05 for all tests.

#### 2.5.1. Comparison with Camera-Based Measurements

To evaluate the accuracy and reliability of the two GNSS-based methods (Method 1 and Method 2), we compared their *SL*, *SF*, and per-step *running velocity* estimates with those obtained from the camera-based measurements. Due to the limited number of steps per subject and track section which could be fully captured within the calibrated areas, data were pooled across both subjects, both legs, and both track sections. For the *SF* (*n* = 20; 2 subjects × 2 sections × 5 steps), five central steps per subject per section were analyzed to minimize edge effects, while the *SL* and *velocity* analyses (*n* = 16; 2 subjects × 2 sections × 4 steps) focused on steps entirely within the calibrated zones. This difference in sample size reflected the spatial constraints of the calibrated areas. We computed the root mean square error (*RMSE*), mean error (*bias*) with 95% confidence intervals (CIs), SD of differences, Pearson’s correlation coefficient (*R*), and the intraclass correlation coefficient (ICC). Bland–Altman plots were generated to evaluate agreement and identify systematic biases, while scatter plots were used to visualize the relationships between the GNSS- and camera-derived measures. The strength of the correlations was interpreted according to the following guidelines [42]. Pearson correlation coefficients (*R*) of 0.90 and above were considered excellent, 0.70–0.89 were considered strong, 0.50–0.69 were considered moderate, 0.30–0.49 were considered weak, and below 0.30 were considered negligible. The reliability assessed by the ICC was interpreted based on the following criteria [43]. ICC values less than 0.50 indicate poor reliability, values between 0.50 and 0.75 indicate moderate reliability, values between 0.75 and 0.90 indicate good reliability, and values greater than 0.90 indicate excellent reliability.

#### 2.5.2. The 400 m Sprint Analysis

For the full 400 m sprint, GNSS-derived step counts were verified against the bicycle-mounted camera, which tracked the total steps taken throughout the sprint. Additionally, the GNSS-derived total sprint time was calculated by determining when the cumulative horizontal distance reached 400 m. This value was compared to the finish-line time recorded by the stationary 2D camera to assess accuracy. Percentage match was used as the evaluation metric, providing a quantitative measure of agreement between the GNSS-derived and reference sprint times.

To further analyze the *SF*, we compared the GNSS-derived *SF* estimates with those obtained from IMU data across the entire 400-m distance. Data were separated for Subject A and Subject B to account for individual differences in running characteristics. We also divided the *SF* data by left and right legs to detect potential asymmetries in the *SF*, using IMU-derived IC events as the reference standard. Furthermore, we investigated the effects of track geometry by analyzing distinct straight (125–195 m and 325–400 m) and curved (5–115 m and 205–315 m) sections, while transitional zones were excluded to maintain clean comparisons. Statistical metrics, including the *RMSE*, *bias* with 95% CI, SD, *R*, and ICC, were computed. Agreement between methods was visually assessed using Bland–Altman plots and scatter plots.

To illustrate dynamic changes in the running parameters throughout the sprint, we visualized the GNSS-derived *SF*, *SL*, and *running velocity* for each step across the entire 400-m distance. These step-by-step visualizations demonstrate how the system can capture every stride variation and highlight performance-relevant factors, such as the peak velocity, changes in *SL*, and gradual decline in running velocity as fatigue sets in. Furthermore, by marking periods of reduced GNSS positional accuracy (i.e., ‘Float’ solutions), we enhanced transparency regarding data quality. This approach underscores the method’s potential utility for detailed training analyses, enabling coaches and athletes to pinpoint stride-by-stride mechanics which were previously averaged over larger intervals.

## 3. Results

### 3.1. Comparison Results with Camera-Based Measurements

During the 3D video analysis sections, both subjects maintained a ‘Fix’ solution throughout, ensuring high positional accuracy for the GNSS measurements. The estimates of the *SF*, *SL*, and per-step *running velocity* obtained from the two GNSS-based methods—Method 1 and Method 2—were compared with the camera-based measurements for the calibrated sections of the track. Data from both subjects were combined for analysis. The statistical metrics for these comparisons are summarized in Table 1.

For the *SF*, both methods showed strong agreement with the camera-based values, with Method 2 exhibiting lower *RMSE* and *bias* and higher ICC and *R* values than Method 1. Similar trends were observed for the *SL*, where Method 2 demonstrated improved accuracy, reflected by lower *RMSE* and *bias* values, along with better consistency across subjects. Regarding the per-step *running velocity*, both methods exhibited excellent agreement with the camera-based measurements, as indicated by high ICC and *R* values.

**Figure 3:**
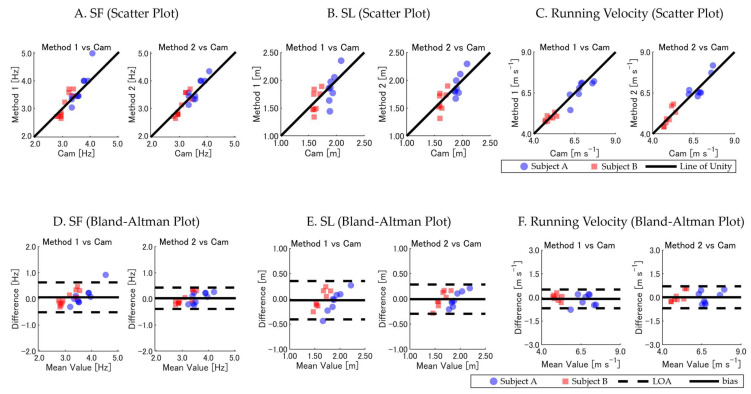
Comparison of GNSS-based methods (Method 1 and Method 2) versus camera-based measurements of *SF*, *SL*, and *running velocity*. (**A**–**C**) Scatter plots with the line of unity. (**D**–**F**) Bland–Altman plots showing *bias* and LOA. Data points are color-coded by subject: Subject A (blue circles) and Subject B (red squares). Overlapping data points appear darker due to transparency settings, visually indicating areas of higher data density.

Visual representations of these comparisons are provided in Figure 3. The scatter plots (panels A, B, and C) demonstrate strong agreement between the GNSS-based estimates and camera-based measurements, with data closely aligning along the unity line. The Bland–Altman plots (panels D, E, and F) reveal that Method 2 exhibited smaller biases and narrower limits of agreement (LOAs) than Method 1 for the *SF* and *SL*. For the *running velocity*, both methods showed comparable performance with minimal differences.

### 3.2. The 400 m Sprint Analysis Results

In the 400 m sprint analysis, Subject A experienced approximately 50% of the data in ‘Float’ mode, whereas Subject B maintained a ‘Fix’ solution for the entire duration. Both GNSS-based methods accurately captured all steps, matching 100% with the reference step counts obtained from the bicycle-mounted camera. Subject A completed 200 steps, and Subject B completed 242 steps. The total sprint times measured by the 2D camera were 55.06 s for Subject A and 73.07 s for Subject B. The GNSS-derived times were 55.090 s for Subject A and 73.065 s for Subject B, showing a 99.95% match for Subject A and 99.99% match for Subject B with the camera-based measurements.

The two GNSS-based methods were evaluated for accuracy in *SF* estimation during the full 400 m sprint, using the IMU-derived *SF* as the reference. Across the trials, the *MAE* and SD for the IMU-based IC detection were 0.005 ± 0.003 s compared with the camera-based reference (*n* = 24), confirming the validity of our method. The results were further analyzed for differences between the subjects, legs (left versus right), and track sections (straight versus curved). Table 2 summarizes the results, including both overall and leg-specific comparisons. For Subject A, Method 2 demonstrated improved accuracy and reliability compared with Method 1, showing a lower *RMSE* (0.232 Hz versus 0.397 Hz), smaller *bias* (0.016 Hz versus 0.058 Hz), and higher ICC (0.711 versus 0.569). When analyzing the left and right legs separately, Method 2 maintained better consistency, with reduced errors and smaller variability. In contrast, Method 1 exhibited larger biases, particularly for the right leg. For Subject B, Method 2 similarly outperformed Method 1, with an *RMSE* of 0.197 Hz compared with 0.219 Hz, a smaller *bias* of 0.017 Hz (versus 0.022 Hz), and a higher ICC of 0.833 compared with 0.809. Method 2 also demonstrated consistent performance for both the left and right legs, whereas Method 1 displayed slightly larger errors and variability in the left leg.

**Figure 4:**
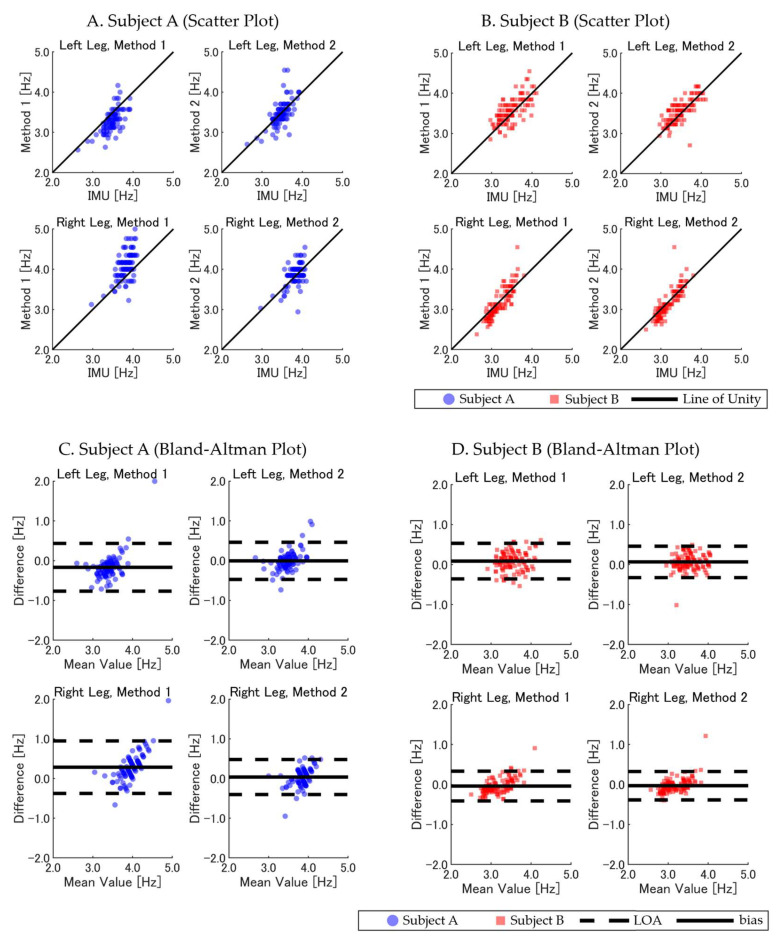
Comparison of GNSS-based methods (Method 1 and Method 2) versus IMU-derived *SF* in the 400 m sprint (left and right legs). (**A**,**B**) Scatter plots for Subject A (blue circles) and Subject B (red squares), with the *SF* for the left leg (**top**) and right leg (**bottom**) compared with the IMU-derived *SF*. (**C**,**D**) Corresponding Bland–Altman plots, indicating *bias* (solid line) and LOA (dashed lines). Overlapping data points appear darker due to transparency settings, visually indicating areas of higher data density.

Figure 4A–D further illustrates the relationships and agreement between the GNSS-based methods and IMU-derived *SF* through scatter plots and Bland–Altman analyses. Method 2 showed stronger alignment with the reference values, with smaller differences and narrower LOAs across both subjects and legs.

Further analysis compared the *SF* estimates in straight and curved track sections across the two GNSS-based methods. The statistical metrics summarized in Table 3 show that Method 2 consistently outperformed Method 1 in terms of accuracy and reliability across both track sections and subjects. For curved sections, Method 2 achieved a lower *RMSE* (0.211 Hz versus 0.348 Hz overall) and *bias* (0.017 Hz versus 0.050 Hz), with higher ICC values (0.835 versus 0.692) compared with Method 1. Similarly, on straight sections, Method 2 demonstrated improved accuracy, with an *RMSE* of 0.168 Hz versus 0.260 Hz for Method 1, along with smaller *bias* (0.012 Hz versus 0.026 Hz) and stronger ICC values (0.858 versus 0.746).

**Figure 5:**
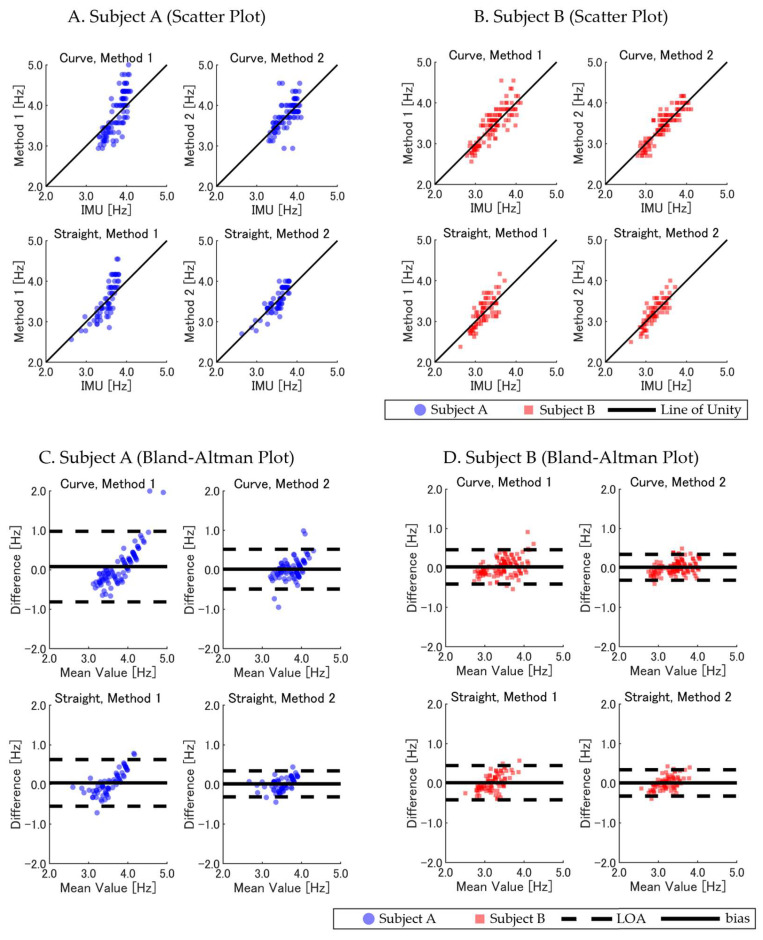
Comparison of GNSS-based methods (Method 1 and Method 2) with IMU-derived *SF* across curved and straight track sections. (**A**,**B**) Scatter plots for Subject A (blue circles) and Subject B (red squares), with data separated into curved (**top**) and straight (**bottom**) sections. (**C**,**D**) Bland–Altman plots indicating *bias* (solid line) and LOA (dashed lines). Overlapping data points appear darker due to transparency settings, visually indicating areas of higher data density.

Figure 5A–D illustrates the relationship and agreement between the GNSS-based methods and IMU-derived *SF*, highlighting the superior performance of Method 2 through tighter clustering around the reference values and narrower LOAs in the Bland-Altman plots.

**Figure 6:**
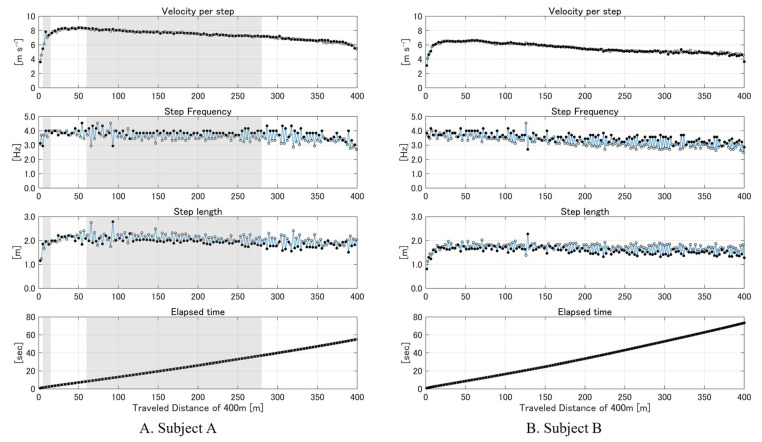
Step-by-step analysis of running parameters using RTK GNSS (Method 2) in a 400 m sprint. Panels show step-by-step changes in *running velocity*, *SF*, *SL*, and *elapsed time* for (**A**) Subject A (200 steps) and (**B**) Subject B (242 steps). The right steps are represented by filled circles (●), and the left steps are represented by open circles (○). Shaded areas for Subject A indicate periods in ‘Float’ solution, highlighting reduced GNSS accuracy.

The running parameters over the entire 400 m sprint were analyzed using Method 2. Due to its superior accuracy and reliability in prior assessments, Method 2 was selected for comprehensive monitoring. Using the vertical velocity minima identified by Method 2, we calculated the *SF*, *SL*, *running velocity*, and elapsed time for each step. These metrics were plotted against the cumulative distance to visualize the running parameters over time (Figure 6). This per-step analysis captured the dynamic changes in *velocity*, *SF*, and *SL* throughout the sprint, providing detailed insights beyond traditional average-based methods. Both subjects exhibited dynamic changes in *SF* and *SL* throughout the sprint. Subject A reached a peak *running velocity* of 8.4 m s^−1^ at the 50.2 m point (*SL* of 2.10 m and *SF* of 4.00 Hz at 7.13 s), while Subject B achieved a peak velocity of 6.6 m s^−1^ at the 63.4 m point (*SL* of 1.86 m and *SF* of 3.57 Hz at 10.75 s). Subsequently, a gradual decline in *running velocity* was observed for both subjects as the sprint progressed. For Subject A, periods during which the GNSS solution shifted from ‘Fix’ to ‘Float’ are highlighted with shaded areas in Figure 6A, indicating reduced positional accuracy.

## 4. Discussion and Implication

### 4.1. Comparison of Methods

The primary aim of this study was to evaluate the accuracy and reliability of two GNSS-based methods—Method 1 (vertical position minima) and Method 2 (vertical velocity minima)—for estimating per-step running parameters, including the *SF*, *SL*, and *running velocity*, during a 400 m sprint. By comparing these two methods with reference standards (3D video analysis and IMU data), we sought to determine which method provides more precise measurements, thereby optimizing the use of RTK GNSS technology in running performance analysis.

Our results demonstrate that both GNSS-based methods could estimate the running parameters. However, Method 2 consistently outperformed Method 1 in *SF* and *SL* estimation across all evaluation criteria. For instance, in the calibrated camera sections, Method 2 exhibited lower *RMSE* values and higher ICCs compared with Method 1 for both the *SF* (0.205 Hz versus 0.291 Hz; ICC: 0.886 versus 0.813) and *SL* (0.143 m versus 0.190 m; ICC: 0.755 versus 0.615). These findings highlight the robustness of Method 2 for precise per-step analyses, particularly in capturing the *SF* and *SL* with greater accuracy. In contrast, for the running velocity, both methods exhibited comparable accuracy, with similar *RMSE* and ICC values. This comparable performance is likely due to the fact that the running velocity, being a product of the *SF* and *SL*, reflects a combination of their respective errors, which may compensate for each other during calculations.

The superior performance of Method 2 can be attributed to its alignment with key biomechanical events. By identifying the minima in the vertical velocity profile of the runner’s head, Method 2 effectively captured the moments of IC. This approach is sensitive to the impact forces during ground contact, analogous to detecting the damping effect in the spring-mass model of running [21,26]. Consequently, Method 2 provided more precise temporal markers for each step, leading to accurate *SF* and *SL* estimations. In contrast, Method 1 relied on detecting the minima in the vertical position of the head at the mid-stance phase. This method is potentially susceptible to left-right asymmetries in leg mechanics, which can cause inconsistencies in vertical displacement patterns [24,25]. Such asymmetries may result in variations in mid-stance timing between the left and right legs, leading to *biases* in *SF* and *SL* estimations. As a result, although the *R* values were similar between Methods 1 and 2, the presence of *bias* in Method 1 led to lower ICC values, indicating reduced reliability. The influence of the track geometry also amplified these differences. Curved sections introduce biomechanical variations [31], such as centripetal force and altered stride patterns, which likely exacerbate the inaccuracies of Method 1. Method 2, however, maintained robustness under these conditions due to its focus on IC events rather than vertical position patterns, making it less prone to the effects of asymmetries.

While Method 2 demonstrated superior accuracy for *SF* and *SL* estimations, both methods showed comparable performance in estimating the *running velocity*. This finding suggests that Method 1 may still be suitable for analyses where the *running velocity* is the primary parameter of interest, although Method 2 provides a more detailed and accurate assessment overall.

### 4.2. Accuracy Achieved, Advantages, and Practical Implications

This pilot study demonstrates the feasibility and potential benefits of using a single RTK GNSS receiver to capture step-by-step running parameters in an outdoor environment. By validating the proposed methods against well-established reference systems (3D video and IMUs), we highlight the possibility of obtaining high-resolution data with minimal drift over an entire 400 m sprint. Although we tested only two collegiate athletes, these findings establish a foundation for larger-scale investigations and support the practical application of RTK GNSSs in regular training environments. The high sampling rate and positional accuracy of the RTK GNSS enabled precise estimation of the *SF*, *SL*, and *running velocity*, even at the high speeds characteristic of sprinting events. The RTK GNSS provided centimeter-level accuracy in horizontal positioning, with a reported precision well below 1 cm [18]. This level of precision is critical for capturing the minute variations in stride parameters which occur during high-speed sprints.

Our findings suggest that the RTK GNSS overcomes the limitations associated with traditional wearable sensors like IMUs and insole sensors, which often face challenges due to cumulative sensor errors and drift over time, especially during high-speed movements [14,16]. Specifically, in the track segments where 3D camera analysis was conducted (the curved and straight sections), the GNSS-based measurements of the *SF* and *SL* showed strong agreement with the camera-based measurements (Table 1, Figure 3A–F). Over the entire 400 m sprint, the GNSS-derived total times closely matched the times recorded by the 2D camera, demonstrating minimal cumulative error, with a 99.95% match for Subject A and a 99.99% match for Subject B. These results indicate that the RTK GNSS maintained consistent accuracy throughout the 400 m distance and effectively addressed the drift issues commonly encountered with IMUs. Unlike IMUs, which can suffer from error accumulation, leading to inaccurate distance measurements over short distances, the RTK GNSS provided stable measurements for both the *SF* (through precise identification of IC timing) and distance covered (*SL* and total cumulative distance). This dual capability means that a single GNSS unit can measure timing events (like an IMU) and accurately quantify distance without complex calibration procedures or data processing algorithms [17].

By measuring the *SL* at high velocities, accurately detecting foot contact events, and consistently measuring the cumulative distance over an entire sprint, the RTK GNSS approach underscores its potential for robust and reliable performance analysis in real-world training. The absence of drift over the 400 m distance allowed for precise, continuous, and detailed per-step analysis, as illustrated in Figure 6, which captures dynamic changes in running parameters beyond traditional average-based methods [6]. This detailed insight offers significant advantages in optimizing training programs and enhancing athletic performance by providing timely information on running mechanics, fatigue effects, and potential biomechanical asymmetries.

Compared with prior research utilizing low sampling rate devices [19], our single high sampling rate (100 Hz) RTK GNSS approach effectively overcomes the limitations of IMU-based systems, such as cumulative errors and drift [14,15,16]. This high-frequency GNSS enables the precise calculation of running parameters, including the *SL* and running velocity, on a per-step basis throughout the entire 400 m sprint, which are capabilities not achievable with existing wearable solutions [9,10,11,13]. By reducing system complexity and eliminating the need for additional devices like IMUs, our method offers a simplified, drift-free solution with a high sampling rate, thereby enhancing both the practicality and accuracy of stride analysis in real-world outdoor training environments.

### 4.3. Study Limitations and Future Challenges

A primary limitation of this study is the small sample size, consisting of only two collegiate athletes. As a pilot study, the aim was to initially evaluate the feasibility and accuracy of the RTK GNSS methods. Consequently, the limited sample may not represent the broader population of 400 m sprinters, and the performance levels of the subjects (55.06 s and 73.07 s for the 400 m sprint) could influence the outcomes. Athletes with different skill levels or running styles may exhibit varying biomechanical patterns, potentially affecting the generalizability of the findings. Future research should include a larger and more diverse cohort of athletes encompassing various skill levels, genders, and training backgrounds to validate the methods across different populations and competitive contexts. 

The GNSS antenna was securely attached to the rugby headgear using adhesive tape, and the headgear itself was firmly fastened to the athlete’s head to minimize relative motion between the head and the antenna. This set-up provided a stable platform for signal reception; however, slight movements of the headgear relative to the athlete’s head may have still occurred at high running speeds, potentially leading to minor measurement errors. While the participants reported no significant discomfort while running, indicating the suitability of this system for training applications which require detailed step-by-step analysis, its practicality for competitive races remains limited. Future refinements, such as improved fixation methods or alternative sensor placements—such as a lightweight back-mounted set-up—could further enhance usability for both training and competition. Additionally, continued miniaturization of GNSS devices may further optimize their application in high-performance sprinting environments.

Additionally, while all trials in the 3D video analysis sections maintained a ‘Fix’ solution, indicating optimal GNSS accuracy, Subject A experienced approximately 50% ‘Float’ solution periods during the 400 m sprint. ‘Float’ solutions indicate reduced positional accuracy due to unresolved integer ambiguities, often influenced by satellite signal conditions and the measurement environment [37]. These periods of reduced accuracy could impact the reliability of the running parameter estimates. Future studies should explore techniques to minimize ‘Float’ solution periods, such as enhancing satellite signal reception in environments with minimal obstructions and incorporating advanced correction algorithms like those proposed by Suzuki [44] to improve the overall accuracy of GNSS measurements.

Furthermore, integrating RTK GNSS data with additional sensor technologies, such as IMUs, could enhance the precision of IC timing detection. While IMUs are susceptible to drift, they offer superior accuracy in detecting contact and toe-off events compared with a GNSS alone. Sensor fusion methods may allow for more accurate identification of IC and toe-off events, enabling calculations of the stance time, flight time, and other detailed temporal parameters. This integration could capitalize on both technologies’ strengths—RTK GNSS for accurate spatial measurements and IMUs for precise temporal event detection—thereby enhancing running parameter estimations’ overall reliability and robustness.

## 5. Conclusions

This study aimed to evaluate the accuracy and reliability of two RTK GNSS-based methods—Method 1 (vertical position minima) and Method 2 (vertical velocity minima)—for estimating the *SF*, *SL*, and *running velocity* in a 400 m sprint. The results demonstrated that both GNSS-based methods successfully estimated the *SF*, *SL*, and *running velocity*, but Method 2 exhibited superior accuracy and reliability. In the 3D video analysis sections, Method 2 showed lower RMSEs and higher ICC values compared with Method 1. Similarly, in IMU-based comparisons across the full 400 m sprint, Method 2 achieved better agreement with the IMU-derived *SF*, particularly in curved sections and in the analysis of left-right asymmetry, where Method 1 exhibited larger biases. The RTK GNSS provided stable measurements throughout the entire 400 m sprint, with the total sprint time matching the 2D camera reference by over 99.95%, confirming its robustness for continuous monitoring. These findings indicate that a single RTK GNSS receiver, particularly when employing the vertical velocity minima approach, enables accurate step-by-step analysis of sprinting performance in outdoor conditions.

## Figures and Tables

**Table 1:** Statistical comparison of GNSS-based methods with camera-based measurements. Note: *n* = 20 for *SF*; *n* = 16 for *SL* and *running velocity*. A double asterisk (**) denotes statistical significance at *p* < 0.01.

Parameter	Method	*RMSE*	*bias*	SD	ICC [95% CI]	*R*
Step Frequency [Hz]	Method 1	0.291	0.061	0.292	0.813 [0.701, 0.926]	0.909 **
	Method 2	0.205	0.025	0.208	0.886 [0.817, 0.955]	0.922 **
Step Length [m]	Method 1	0.190	−0.027	0.194	0.615 [0.349, 0.882]	0.681 **
	Method 2	0.143	−0.006	0.148	0.755 [0.585, 0.925]	0.799 **
Running Velocity [m s^−1^]	Method 1	0.308	−0.094	0.303	0.951 [0.917, 0.985]	0.959 **
	Method 2	0.344	0.010	0.355	0.950 [0.916, 0.985]	0.949 **

**Table 2:** Comparison of *SF* (Hz) estimates from GNSS-based methods with IMU data by subject and leg. Note: A double asterisk (**) denotes statistical significance at *p* < 0.01.

Subject	Leg	Method	*RMSE*	*bias*	SD	ICC [95% CI]	*R*
A	Both	Method 1	0.397	0.058	0.393	0.569 [0.482, 0.655]	0.762 **
		Method 2	0.232	0.016	0.232	0.711 [0.652, 0.769]	0.748 **
	Left	Method 1	0.348	−0.167	0.307	0.306 [0.109, 0.503]	0.544 **
		Method 2	0.237	−0.005	0.238	0.600 [0.486, 0.714]	0.661 **
	Right	Method 1	0.440	0.284	0.337	0.115 [−0.136, 0.367]	0.544 **
		Method 2	0.227	0.037	0.225	0.489 [0.343, 0.634]	0.545 **
B	Both	Method 1	0.219	0.022	0.218	0.809 [0.774, 0.843]	0.845 **
		Method 2	0.197	0.017	0.196	0.833 [0.803, 0.863]	0.855 **
	Left	Method 1	0.241	0.085	0.227	0.639 [0.548, 0.731]	0.705 **
		Method 2	0.210	0.066	0.200	0.712 [0.638, 0.785]	0.754 **
	Right	Method 1	0.193	−0.040	0.190	0.800 [0.749, 0.851]	0.878 **
		Method 2	0.183	−0.031	0.181	0.807 [0.758, 0.856]	0.862 **

**Table 3:** Comparison of *SF* estimates from GNSS-based methods with IMU data by subject and track section. Note: A double asterisk (**) denotes statistical significance at *p* < 0.01.

Subject	Section	Method	*RMSE*	*bias*	SD	ICC [95% CI]	*R*
A	Curve	Method 1	0.461	0.080	0.457	0.458 [0.304, 0.612]	0.710 **
		Method 2	0.257	0.016	0.258	0.599 [0.485, 0.713]	0.648 **
	Straight	Method 1	0.301	0.040	0.300	0.665 [0.551, 0.779]	0.817 **
		Method 2	0.167	0.015	0.168	0.824 [0.764, 0.884]	0.856 **
B	Curve	Method 1	0.223	0.026	0.222	0.826 [0.782, 0.869]	0.854 **
		Method 2	0.167	0.017	0.167	0.892 [0.865, 0.919]	0.906 **
	Straight	Method 1	0.219	0.015	0.220	0.717 [0.628, 0.806]	0.782 **
		Method 2	0.169	0.009	0.169	0.805 [0.744, 0.866]	0.843 **
All	Curve	Method 1	0.348	0.050	0.345	0.692 [0.634, 0.750]	0.782 **
		Method 2	0.211	0.017	0.211	0.835 [0.804, 0.866]	0.852 **
	Straight	Method 1	0.260	0.026	0.259	0.746 [0.687, 0.805]	0.825 **
		Method 2	0.168	0.012	0.168	0.858 [0.826, 0.891]	0.881 **

## Data Availability

The datasets generated and analyzed during the current study are available from the corresponding author upon reasonable request.
